# Microwave-Assisted Solution-Phase Synthesis and DART-Mass Spectrometric Monitoring of a Combinatorial Library of Indolin-2,3-dione Schiff Bases with Potential Antimycobacterial Activity

**DOI:** 10.3390/molecules16065194

**Published:** 2011-06-22

**Authors:** Tarek Aboul-Fadl, Hatem A. Abdel-Aziz, Adnan Kadi, Pervez Ahmad, Tilal Elsaman, Mohamed W. Attwa, Ibrahim A. Darwish

**Affiliations:** Department of Pharmaceutical Chemistry, College of Pharmacy, King Saud University, P.O. Box 2457, Riyadh 11451, Saudi Arabia

**Keywords:** combinatorial library, Schiff bases, solution-phase synthesis, microwave irradiation, indolin-2,3-dione, DART, antimycobacterial activity

## Abstract

A combinatorial library composed of eleven hydrazides **A-K **and eleven indolin-1,2-dione derivatives **1-11** has been designed to formally generate sublibraries of 22 mixtures, **M_1_-M_22_** comprising of 121 Schiff bases, **A-K**(**1-11**). The designed library has been synthesized by the solution-phase method and microwave-assisted synthetic techniques. The formation of individual compounds of each mixture was confirmed by Direct Analysis in Real Time (DART) as ionization technique connected to an Ion Trap as a mass detector. The synthesized mixtures were evaluated for their antimycobacterial activity against four *Mycobacterium* strains; *M. intercellulari, M. xenopi, M. cheleneoi* and *M. smegmatis*. Variable antimycobacterial activity was revealed with the investigated mixtures and maximum activity was shown by **M_8_**, **M_10_**, **M_11_**, and **M_15_** with MIC values of 1.5, 3.1, 6.2 and 0.09 μg/mL, respectively. Application of the indexed method of analysis on these active mixtures revealed that compounds **D_8_**, **D_10_** and **D_11_** may contribute to the activity of the tested mixtures.

## 1. Introduction

Tuberculosis (TB) is again becoming a serious problem worldwide, and is responsible for at least three million deaths annually, as a life is lost to TB every 15 seconds [1,2]. Factors involved in the resurgence of TB [3] include the AIDS epidemic, which emerged in the mid-1980s, and the rapid spread of the multidrug-resistant (MDR) TB strain which is unaffected by the major anti-tuberculosis drugs currently on the market [4]. Particularly worrisome is the super bacterium XDR-TB (extensively drug-resistant tuberculosis), which is resistant to all first and second line anti-TB drugs [5]. Thus there is an urgency to develop new drugs and strategies to fight against tuberculosis or a tragedy may occur.

Due to their synthetic and biological versatility, hydrazones are attractive target compounds for new drug development. In literature, many pharmacological properties involving antitubercular activity have been associated with hydrazones [6,7,8,9,10,11]. Furthermore, there are significant reasons for investigating Schiff base derivatives of indolin-2,3-dione (isatin); one of these reasons is the recent reports of their remarkable anti-TB activity [12,13,14,15]. Other reasons that can be mentioned are their significant lipophilicities, which could facilitate their entry into the intracellular environment and the carbohydrate nature of the cell wall of *Mycobacterium tuberculosis* [16].

Technological advances have provided a driving force for the rapid evolution of the drug discovery process [17]. Combinatorial chemistry has been one of the most rapidly developing fields in the pharmaceutical industry in recent years. It is now an essential tool, both in the discovery and the development of new drugs. The advent of this chemistry techniques, have altered the face of medicinal chemistry forever and a very substantial literatures has been developed in a short time. Thus, the generation and use of combinatorial chemical libraries for the identification of novel chemical leads or for the optimization of a promising lead candidate has emerged as a potentially powerful method for the acceleration of the drug discovery process [18,19]. However, there are a few reports on solution phase combinatorial synthesis and combinatorial antibacterial screening, despite the simplicity, time and material saving factors involved in the process [20,21,22]. Additionally, microwave synthesis represents a major breakthrough in synthetic chemistry methodology, dramatic changes in the way chemical synthesis is preformed and in the way it is perceived in the scientific community. Over the last few years, there has been growing interest in the synthesis of organic compounds under green chemistry such as microwave irradiation because of increasing environmental consciousness. The feasibility of microwave assisted synthesis has been demonstrated in various transformations. The features of these transformations are: enhanced reaction rate, greater selectivity and experimental ease of manipulation leading to an efficient, environmental friendly beside cost effective synthesis pathway of several compounds [23,24,25,26]. Moreover, the use of microwave irradiation in this regard is now a well-established procedure in MORE (microwave induced organic reaction enhancement) chemistry [27]. Additionally, direct analysis in real time (DART) is a novel ionization technique that provides for the rapid ionization of small molecules under ambient conditions. DART is based on the reactions of electronic or vibronic excited-state species with reagent molecules and polar or non-polar analytes. Although DART has been applied to the analysis of gases, liquids, and solids, a unique application is the direct detection of chemicals on surfaces without requiring sample preparation, such as wiping or solvent extraction. DART has demonstrated success in sampling hundreds of chemicals, including chemical agents and their signatures, pharmaceutics, metabolites, peptides and oligosaccharides, synthetic organics, organometallics, drugs abuse, explosives, and toxic industrial chemicals. These species were detected on various surfaces, such as concrete, asphalt, human skin, currency, airline boarding passes, business cards, fruits, vegetables, spices, beverages, body fluids, horticultural leaves, cocktail glasses, and clothing. DART employs no radioactive components and is more versatile than devices using radioisotope-based ionization. Because its response is instantaneous, DART provides real-time information, a critical requirement for screening or high throughput. It can be used as a means to quickly monitor synthetic organic reactions and to obtain nearly instantaneous molecular weight confirmations of final products in drug discovery [28,29]. 

Accordingly, as a contribution to the anti-TB drug development using combinatorial chemistry and microwave assisted synthesis technologies utilizing the advantages of DART technology for mixtures components monitoring, the current work describes design, solution- phase combinatorial synthesis of a mixture-based Schiff base library. The combinatorial screening will lead to expeditious identification of structures which could be novel leads for antimycobacterial activity optimization [30].

## 2. Results and Discussion

### 2.1. Chemistry

Combinatorial libraries have been rapidly accessed using a variety of methodologies and techniques that have been developed for use in solid-phase, as well as solution-phase synthesis. Although solid-phase has been at forefront of combinatorial chemistry, parallel solution-phase synthesis is an interesting alternative approach. The advantages that characterize solution-phase synthesis include validation time, facility of manipulations and the diversity of reactions that can be performed [31]. A solution-phase library approach is an attractive choice if reactions provided high yield production and removable byproducts [32]. The current mixture-based combinatorial library was designed in accordance with literatures survey for antimycobacterial activity of Schiff bases of isatin derivatives [12,15]. A two-component coupling reaction for library synthesis was selected. This is the simplest reaction type upon which combinatorial libraries have been based and it has been applied widely [33]. Hydrazone formation was also used as the base of library synthesis as it generates only water as a by-product. The current library is composed of eleven isatins **1-11** and eleven hydrazides **A-K** to generate 121 hydrazones as shown in the matrix displayed in Table 1. 

Cells in the library displayed by the 11 × 11 matrix serve as visual aids in determining contents of each mixture and in deconvoluting them. The first column and row represents the starting materials, and the rest represents the synthesized compounds. The matrix describes also 22 mixtures produced while the last column and row represent mixtures **M_1_-M_11_** and **M_12_-M_22_**, respectively.

**Scheme 1:**
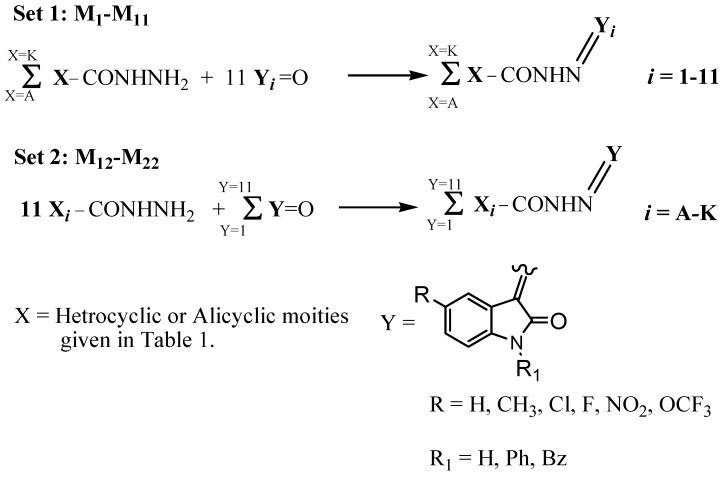
Synthesis of the sub-library sets 1 and 2.

As depicted in Scheme 1, each of the mixtures **M_1_-M_11_** of set 1 was prepared by reacting each of isatins **1-11**, in an ethanolic solution containing a catalytic amount of glacial acetic acid, with the 11 hydrazides **A-K** in equimolar amounts under microwave irradiation (200 W, 110 °C) for 10 min. Each mixture **M_1_-M_11 _**thus produced consists of eleven Schiff bases, **1**(**A-K**), **2**(**A-K**), **3**(**A-K**), **4**(**A-K**), **5**(**A-K**), **6**(**A-K**), 7(**A-K**), **8**(**A-K**), **9**(**A-K**), **10**(**A-K**) and **11**(**A-K**). It is worthy to mention that each mixture **M_1_-M_11_** has the reaction products of the fixed isatin **1-11** and all 11 hydrazides **A-K**. In the same way, the second set of the sub-library **M_12_-M_22_** was prepared, however, under the same previous conditions for mixtures **M_1_-M_11_** by reacting each of hydrazides **A-K**, with the all 11 isatins **1-11** in equimolar amounts. Each **M_12_-M_22_** mixture produced consists of eleven Schiff bases, **A**(**1-11**), **B**(**1-11**), **C**(**1-11**), **D**(**1-11**), **E**(**1-11**), **F**(**1-11**), **G**(**1-11**), **H**(**1-11**), **I**(**1-11**), **J**(**1-11**) and **K**(**1-11**). Combining information coming from the biological screening of the mixtures by orthogonal intersection of the two sets of sub-library, the best individual compound can be identified.

Assignments of the structures of the synthesized combinatorial library were done on basis of the data obtained by IR and DART mass spectrometric techniques. The expected carbonyl group absorption bands were observed at 1650–1690 cm^−1^. The various substituent groups on the isatins and hydrazides used for the synthesis of the respective sub-libraries exhibited bands at 745–755 (C-Cl), 1050–1055 (C-O), 1610–1620 (NO_2_), 2800–2900 (CH, aliphatic), 3000–3050 (CH, aliphatic) and 3200–3350 (NH) cm^−1^. 

The synthesized combinatorial mixtures were also studied for molecular ion peaks for compounds employing DART as ionization technique connected to an Ion Trap as a mass detector. The DART spectroscopic method is fast, simple, and can detect trace chemicals in complex matrices, using minimal or no sample preparation. The samples usually states their original chemical/physical/ biological without external interference before ionization. Recently, the DART technique has been used for chemical profiling of the different landraces of *Piper betle* leaves [34], quality and authenticity assessment of olive oil [35], the detection of breast cancer [36], screening for pesticides [37], serum metabolomic fingerprinting [38], identification of multiple mycotoxins in cereals [39], *etc*. To the best of our knowledge, DART has not been used to scan organic small molecules in combinatorial mixtures yet. 

**Table 1:** Building blocks and the designed Schiff bases combinatorial library.

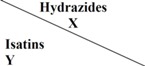		A	B	C	D	E	F	G	H	I	J	K	
		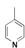	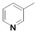	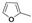	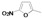	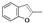	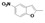	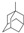	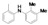	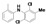	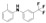	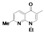	Set. 1
**1**	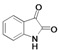	**A_1_**	**B_1_**	**C_1_**	**D_1_**	**E_1_**	**F_1_**	**G_1_**	**H_1_**	**I_1_**	**J_1_**	**K_1_**	**M_1_**
**2**	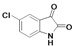	**A_2_**	**B_2_**	**C_2_**	**D_2_**	**E_2_**	**F_2_**	**G_2_**	**H_2_**	**I_2_**	**J_2_**	**K_2_**	**M_2_**
**3**	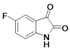	**A_3_**	**B_3_**	**C_3_**	**D_3_**	**E_3_**	**F_3_**	**G_3_**	**H_3_**	**I_3_**	**J_3_**	**K_3_**	**M_3_**
**4**	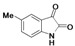	**A_4_**	**B_4_**	**C_4_**	**D_4_**	**E_4_**	**F_4_**	**G_4_**	**H_4_**	**I_4_**	**J_4_**	**K_4_**	**M_4_**
**5**	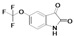	**A_5_**	**B_5_**	**C_5_**	**D_5_**	**E_5_**	**F_5_**	**G_5_**	**H_5_**	**I_5_**	**J_5_**	**K_5_**	**M_5_**
**6**	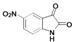	**A_6_**	**B_6_**	**C_6_**	**D_6_**	**E_6_**	**F_6_**	**G_6_**	**H_6_**	**I_6_**	**J_6_**	**K_6_**	**M_6_**
**7**	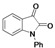	**A_7_**	**B_7_**	**C_7_**	**D_7_**	**E_7_**	**F_7_**	**G_7_**	**H_7_**	**I_7_**	**J_7_**	**K_7_**	**M_7_**
**8**	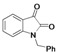	**A_8_**	**B_8_**	**C_8_**	**D_8_**	**E_8_**	**F_8_**	**G_8_**	**H_8_**	**I_8_**	**J_8_**	**K_8_**	**M_8_**
**9**	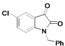	**A_9_**	**B_9_**	**C_9_**	**D_9_**	**E_9_**	**F_9_**	**G_9_**	**H_9_**	**I_9_**	**J_9_**	**K_9_**	**M_9_**
**10**	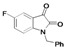	**A_10_**	**B_10_**	**C_10_**	**D_10_**	**E_10_**	**F_10_**	**G_10_**	**H_10_**	**I_10_**	**J_10_**	**K_10_**	**M_10_**
**11**	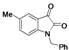	**A_11_**	**B_11_**	**C_11_**	**D_11_**	**E_11_**	**F_11_**	**G_11_**	**H_11_**	**I_11_**	**J_11_**	**K_11_**	**M_11_**
**Set 2**		**M_12_**	**M_13_**	**M_14_**	**M_15_**	**M_16_**	**M_17_**	**M_18_**	**M_19_**	**M_20_**	**M_21_**	**M_22_**	

**Figure 1:**
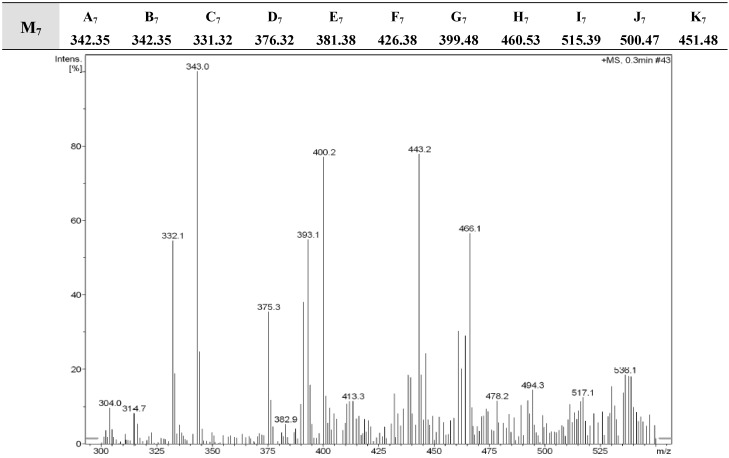
DART-MS of **M_7_**.

**Figure 2:**
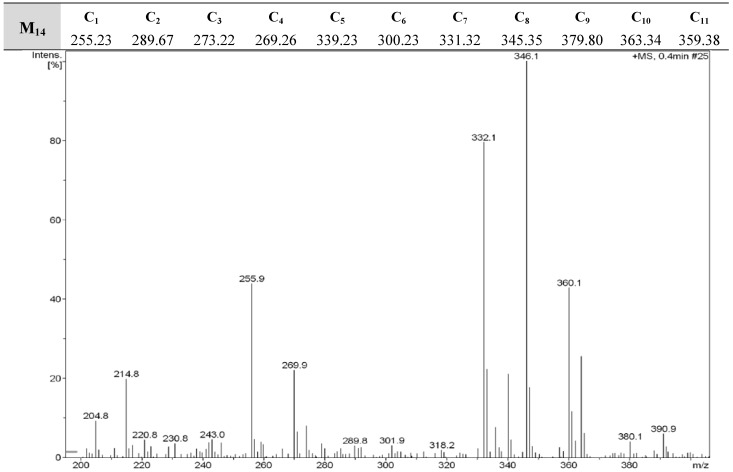
DART-MS of **M_14_**.

The DART-Mass spectrometric technique was used to establish that the expected compounds were indeed produced and present in the synthesized sub-library. The molecular ion peaks obtained from these measurements are a set of data directly correlated to the diversity of a given molecular library. Since fragmentation was not a factor, we were able to compare the molecular ion peaks in the mass spectra with the molecular weights expected for each sub-library, and come to a conclusion regarding which compounds had been formed and which had not. The spectra of the tested mixtures **M_1_-M_22_** showed molecular ion peaks corresponding to the individual compounds of each mixture. This provides strong evidence that all components might be present in sufficient amounts. Representative spectra of the DART-MS analysis of **M_7_** and **M_14_** are given in Figure 1 and Figure 2. 

### 2.2. Antimycobacterial Activity

The 22 mixtures, **M_1_**-**M_22,_** of the synthesized library were tested for their antimycobacterial activity against four *Mycobacterium* strains: *M. intercellulari, M.xenopi, M. cheleneoi* and *M. smegmatis* according to the protocol mentioned in the Experimental section. Control experiments were done using a growth medium free of the investigated compounds and results are shown in Table 2. 

Variable antimycobacterial activity was revealed with the investigated mixtures and maximum activity was shown by **M_8_**, **M_10_**, **M_11_**, and **M_15_**, with MIC values of 1.5, 3.1, 6.2 and 0.09 μg/mL respectively. Surprisingly, other mixtures did not show activity on the tested strains up to concentration of 100 μg/mL.

The indexed method of analysis of the prepared library was applied to elucidate the active components in mixtures. Intersection of the active rows **M_8_**, **M_10_** and **M_11,_** and column **M_15_** in Table 2 allowed the location of the active compound in these mixtures. Accordingly, compounds **D_8_**, **D_10_** and **D_11_** may contribute to the activity of the tested mixtures (Table 2). In order to confirm the reliability of the predictions from the crossing procedure, the synthesis of the individual compounds and their investigation against *Mycobacterium* strains are currently in progress. It is as yet too early to predict the contribution of the various building blocks to the biological activity. However, the resulting activity deduced from the orthogonal method revealed the main contribution of the 5-nitrofuran, 1-benzylisatin and 1-benzyl-5-substitued isatin building blocks on the antimycobacterial activities. This observation is consistent with previously reported data [12,13,15]. 

## 3. Experimental

### 3.1. General

Building blocks **A**-**F**, and **1-7** of the designed library were obtained commercially, however, the other building blocks of the library were synthesized according to the reported literatures [12,14,40,41]. All other chemicals used were of commercially available reagent grade and were used without further purification. Microwave irradiations were carried out using an Explorer-48 microwave reactor (CEM, USA). Infrared (IR) Spectra were recorded as KBr disks using a Perkin Elmer FT–IR Spectrum BX apparatus at the research center, College of Pharmacy, King Saud University, Saudi Arabia. 

**Table 2:** Antimycobacterial evaluation results of the synthesized combinatorial mixtures M_1_-M_22_ and schematic representation of an orthogonal deconvolution for prediction of the active compounds.

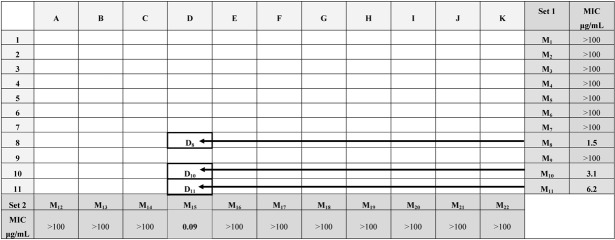

^*^ MIC of the reference drug INH is 12.5 μg/mL under the same experimental conditions.

DART- Mass spectrometric spectra were taken at the Department of Pharmaceutical Chemistry, College of Pharmacy, King Saud University, Saudi Arabia on an a ion trap LC/MS mass spectrometer from Agilent Technologies equipped with an IonSense (Saugus, MA, USA) DART source Antimycobacterial screening was carried out at the Research Center, College of Pharmacy, King Saud University, Saudi Arabia.

### 3.2. Preparation of Combinatorial Mixtures

#### 3.2.1. Synthesis of Schiff base mixtures **M_1_-M_11_** (set. 1)

To a mixture of ethanolic solution (25 mL) of the individual isatins **1-11** (11 mmol) and the mixed hydrazides **A-K** (1 mmol for each) in a closed microwave reactor vessel, glacial acetic acid (1 mL) was added and the reaction mixtures were irradiated under microwave irradiation at 110 °C for 10 minutes at 200 W. The reaction mixture was cooled, the separated solid was filtered, washed with cold ethanol, and dried without further purification to afford mixtures **M_1_-M_11_**.

#### 3.2.2. Synthesis of Schiff base mixtures **M_12_-M_22_** (set. 2)

To a mixture of ethanolic solution (25 mL) of the individual hydrazides **A-K** (11 mmol) and the mixed isatins **1-11** (1 mmol for each) in microwave reactor closed vessel, glacial acetic acid (1 mL) was added and the reaction mixtures were irradiated under microwave irradiation at 110 °C for 10 minutes at 200 W. The reaction mixture was cooled, the separated solid was filtered, washed with cold ethanol, and dried without further purification to afford mixtures **M_12_-M_22_**.

### 3.3. DART-Spectrometric Analysis

The DART source used helium gas at a flow rate of 4 L/min. The gas heater and capillary voltage of the DART source were set to 350 °C, 4000 respectively. The distance between the outlet of the DART gas and the inlet of the orifice of Ion trap LC/MS was 1 cm. Sample introduction was accomplished by slowly moving the closed end of a melting point capillary, which was dipped into a powdered analytes so that the sample was carried across the helium gas stream between the DART source and the orifice of the Ion trap LC/MS. The spectra recording interval was 0.5 s. Each spectrum shown in the figures represents the corresponding spectrum at the maximum of a total ion current (TIC) chromatogram.

### 3.4. Evaluation of Antimycobacterial Activity of the Synthesized Library

The tested Mycobacterium strains are *M. intercellulari* (ATCC 35743)*, M.xenopi* (ATCC 14470)*, M. cheleneoi* (ATCC 35751)*and M. smegmatis* (ATCC 35797) using Rist and Grosset proportion method (agar dilution method) [39].

The mixtures **M_1_-M_12_** and **INH** were solubilized in DMSO at a concentration of 1 mg/mL. An appropriate aliquot of each solution was diluted with 10% molten agar to give concentrations of 100 μg/mL. The agar and the compound solution were mixed thoroughly and the mixture was poured into Petri-dishes on a level surface to result in an agar depth of 3 to 4 mm and allowed to harden. The incula were prepared by growing overnight culture in Muller-Hinton broth. The cultures were diluted 1:100. Tested organisms were streaked in a radial pattern and plates were incubated at 35 °C for 48 hr to check the growth of the tested strains at this single concentration. Active mixtures were further diluted and tested by the same way to determine the minimum inhibitory concentration (MIC) of these mixtures. Experiment using the tested strains in a medium free of investigated compounds was also carried out and the results are given in Table 2.

## 4. Conclusions

A combinatorial library composed of 22 mixtures, **M_1_-M_22_** comprising of 121 Schiff bases, **A-K** (**1-11**) has been synthesized by solution-phase methods and microwave-assisted synthetic techniques. Formation of individual compounds of each mixture was confirmed by the DART mass spectrometric technique. The antimycobacterial evaluation results have provided three lead compounds (**D_8_**, **D_10_** and **D_11_**) with 5-nitrofuran, 1-benzylisatin and 1-benzyl-5-substitued isatin building blocks, however, further exploration of the contribution of the building blocks to obtain potent antimycobacterial agents are currently in progress.
